# Potentially modifiable factors contribute to limitation in physical activity following thoracotomy and lung resection: a prospective observational study

**DOI:** 10.1186/1749-8090-9-128

**Published:** 2014-09-27

**Authors:** Paula J Agostini, Babu Naidu, Pala Rajesh, Richard Steyn, Ehab Bishay, Maninder Kalkat, Sally Singh

**Affiliations:** Department of Thoracic Surgery, Heart of England NHS Foundation Trust, Bordesley Green East, Bordesley Green, Birmingham UK; Birmingham Medical School, University of Birmingham, Birmingham, UK; Centre for Exercise and Rehabilitation Science, Leicester Respiratory Biomedical Research Unit, University Hospitals of Leicester NHS Trust, Glenfield Hospital, Leicester, UK

**Keywords:** Thoracic surgery, Physical activity, Early mobilisation

## Abstract

**Background:**

Early mobility is considered important in minimising pulmonary complication, length of stay (LOS) and enhancing recovery following major surgery. We aimed to observe and measure the reduction in early postoperative physical activity following major thoracic surgery, identifying any potentially limiting factors, and factors predictive of reduced activity.

**Methods:**

Patients undergoing thoracotomy and lung resection were prospectively observed for the purposes of this study. All patients were routinely assisted to mobilise by physiotherapists from postoperative day 1, and continued daily with exercise and progression of mobility as per usual practice. Physical activity was measured with SenseWear Pro 3 armband physiologic motion sensors between postoperative day 1–4. The motion sensors recorded step count, time spent in ‘sedentary’/ ‘moderate’ activity, and energy expenditure. Frequency of postoperative pulmonary complication (PPC) and postoperative LOS were also observed. Multivariate analyses were performed using forward stepwise logistic regression; results are displayed as odds ratio (95% confidence intervals).

**Results:**

n = 99, median (interquartile range) steps 472 (908) over combined postoperative days 2/ 3, sedentary activity (<3 METs) 99%. Less active subjects reported significantly more pain on day 2 and 3 (p = 0.013/ 0.00 respectively) (p < 0.001). On regression analysis age ≥75 years, predicted FEV_1_ < 70% and poor preoperative activity were predictive of lower postoperative activity. Factors limiting mobility on day 1 included pain and dizziness. Median LOS was longer (p = 0.013) (6 vs. 5 days) in less active patients and frequency of PPC was 20% vs 4% (p = 0.034).

**Conclusion:**

Physical activity following major thoracic surgery is generally very limited, with less active patients demonstrating longer LOS. Factors limiting immediate postoperative mobility were largely modifiable, some of the factors predictive of lower activity were also possibly modifiable/amenable to physiotherapy or pulmonary rehabilitation. Prompt assessment and recognition of these factors is needed in future, with timely and effective management incorporated into care pathways to maximise each patients potential to mobilise postoperatively.

**Trial registration:**

ISRCTN52709424

**Electronic supplementary material:**

The online version of this article (doi:10.1186/1749-8090-9-128) contains supplementary material, which is available to authorized users.

## Background

Early mobilisation following thoracotomy and lung resection is frequently undertaken [1] with the purpose of minimising postoperative pulmonary complication (PPC); this type of care is associated with improved outcomes when applied in an enhanced recovery, fast-track [2, 3] or physiotherapy protocol [4]. Observations of physical activity in surgical patients are limited; two studies observe activity preoperatively [5, 6] and one a month postoperatively [6]. Observation of early upright position postoperatively has also shown benefit [7], with limited ‘uptime’ (time spent in the upright position) associated with increased length of stay (LOS) [8]. Despite the belief that early upright position and mobilisation are important following major surgery, the exact amount of physical activity undertaken, and any possible limiting factors, remain undefined.

Reduced physical activity following surgery may be caused by many factors; pain, drowsiness and the addition of surgical attachments. Other possible factors include reduction in quadriceps strength [9], ventilatory impairment [10, 11], and dyspnoea [12] which may lead to decreased exercise tolerance [13]. Any factor that is preventable, reversible or modifiable needs to be identified and addressed within postoperative care pathways, more so given the increasing number of high risk patients undergoing this type of surgery. The main aims of this research were to determine how physically active patients were immediately following major thoracic surgery, and to identify any specific factors contributing to possible limitation.

## Methods

### Design

This prospective, observational study was conducted between October 2008 and October 2010 as part of a single-blind randomised controlled trial investigating effectiveness of deep breathing exercises [14] where no significant differences were detected. Ethical approval for this study was granted in 2008 by the Local Research Ethics Committee (South Birmingham) following approval from the local clinical governance department (REC number H1207/79).

### Participants

The randomised sample was identified from the accessible population in a tertiary, regional thoracic centre. Eligible patients were approached for written consent after screening with inclusion (male/ female patients undergoing planned thoracotomy and lung resection, aged 18 or over, willingness to participate) and exclusion criteria (emergency thoracotomy, procedures involving the mediastinum and chest wall, lung resection via minimally invasive surgery or unplanned progression of this to thoracotomy). Decisions regarding patient operability and resectability were informed by UK national guidelines [15]. Following surgery patients were managed overnight in a thoracic high dependency unit (HDU) (level 2 care), and then on the thoracic surgical ward. Postoperative pain control was initially achieved by continuous thoracic epidural analgesia, intrathecal morphine and/or intercostal blocks or systemic opioids followed by oral analgesia. Intercostal chest drains were managed as per the surgical unit protocol, and included continuous chest wall suction as necessary (to maintain lung expansion). Drains were assessed daily for removal by the surgical team.

### Intervention

Daily physiotherapy was commenced on postoperative day 1. Deep breathing exercises were supervised, and supported coughing was taught. Patients were routinely assisted to mobilise by physiotherapists within the ward area during each physiotherapy session from postoperative day 1 (even if this required 2 or more members of staff) and twice from day 2 onwards. All were initially assisted to mobilise 2 lengths of a 25 m ward area (or equivalent), and this distance was progressed at each session. If subjects were not deemed fit enough to manage this distance (for example due to pain, nausea or breathlessness) they mobilised as far as safely possible.

### Data collection

SenseWear Pro3 armband motion sensors (APC Cardiovascular Ltd, Crewe, UK) were used to measure physical activity. This type of monitor (motion sensor) has been shown to be the most accurate and the most suitable tool for assessing activity in more sedentary patients [16]. Monitors were applied on postoperative day 1 during physiotherapy sessions, and worn until day 4. The device, which was compact (8.8 × 5.6 × 2.1 cm) was mounted on the upper arm with a soft, velcro strap. Data from the SenseWear Pro3 armband motion sensors were obtained using Sensewear Professional software, which calculates time spent in activity of differing intensity as defined by metabolic equivalent of a task (MET) and energy expenditure.

Demographics and potential confounders reflecting potential risk for the development of PPC [17] were recorded. These factors included age, history of chronic obstructive pulmonary disease (COPD), American Society of Anaesthesiologists score (ASA), body mass index (BMI), and smoking status. Postoperative perceived pain scores were recorded by nursing staff, as rated by patients using a score of 0 to 3; 0 indicated no pain, 1 mild pain, 2 moderate pain and 3 severe pain. An 8 point subjective score was also routinely used at the preoperative assessment clinic to describe preoperative activity level; 1) bedbound, 2) wheelchair/bed to chair, 3) 5 m/across a room, 4) 25 m/length of ward, 5) 100 m/length of football pitch, 6) 400 m/distance between bus stops, 7) 2 km/30 minute walk, 8) >2 km/no exercise limitation.

### Outcome measures

Levels of postoperative activity are reported in terms of steps, time spent in ‘sedentary’ (<3 MET) or ‘moderate/ vigorous’ activity (3–6 MET), total energy expenditure, and active energy expenditure (derived from activity >3 MET). Frequency of PPC and postoperative LOS (not including day of surgery) were also observed. PPC was recognised in the presence of 4 or more of the 8 variables [18]; chest x-ray signs of atelectasis/ consolidation, elevated white cell count >11.2 × 10^9^/L or administration of respiratory antibiotics, temperature >38°C, positive signs of infection on sputum microbiology, oxygen saturation < 90% on room air, new/changed purulent sputum production (yellow or green), physician diagnosis of pneumonia or chest infection and re-admission or prolonged stay (over 36 hours) in the intensive care unit /HDU with problems which are respiratory in origin.

### Data analysis

Analysis of data was performed using SPSS Version 17. Normally distributed continuous variables are expressed as mean (±standard deviation), skewed continuous variables as median (interquartile range) and categorical variables as percentages. Differences were tested for as appropriate with Chi-square, Fisher’s exact, independent samples *t*-test and the Mann–Whitney U tests. A p-value <0.05 was considered significant. Multivariate analyses were performed using forward stepwise logistic regression; results are displayed as odds ratio (95% confidence intervals).

## Results

### Flow of patients through the study

Monitors were applied to 147 patients, and 99 had complete, uninterrupted data sets. 43 data sets were not analysed; 32 patients removed the monitor during the study period, 3 were discharged from hospital on postoperative day 3, 3 monitors did not record (no known reason), 3 monitors did not have sufficient battery, 2 patients became too unwell and 4 were excluded as they had reached the end point of PPC when assessed on postoperative day 1. There were no intensive care unit admissions or deaths.

### Demographic and risk factors

46 subjects were male (46%) and 92 (93%) had lung cancer. The mean age was 67 (±10) years, mean percentage predicted forced expiratory volume in one second (FEV_1_) 75% (±19) and mean BMI 26 (±4). 27 patients had a history COPD (27%), 61 ASA score ≥3 (62%), and 22 were current smokers/ ex ≤ 6 weeks (22%). 10 patients underwent pneumonectomy (10%), 57 lobectomy (58%), 19 wedge resection (19%), 4 segmentectomy (4%), 5 exploratory thoracotomy (5%) and 4 sleeve resection (4%). No patient reported preoperative activity score of 1–3. 23 subjects reported restriction of less than 400 m (score 4–6); 13 due to shortness of breath (55%) and 10 orthopaedic, neurological or circulatory comorbidities (45%).

### Postoperative activity

Observed postoperative physical activity is displayed in Table 1. Increase in step count from postoperative day 2 to 3 was significant (p = 0.008). Twenty two subjects were not able to mobilise on postoperative day 1 (22%); 11 due to dizziness secondary to low blood pressure (epidural analgesia noted in 6/11), 2 pain, 2 continuous intercostal drain suction, 2 cardiac instability, 1 vomiting, 2 drowsiness, 1 nausea and 1 orthopaedic co-morbidity.

**Table 1:** **Observed postoperative physical activity**

Physical activity	Postoperative day 2. 8 am-8 pm	Postoperative day 3. 8 am-8 pm	Early postoperative period 8 am postoperative day 2 –8 pm postoperative Day 3
**Median (interquartile range) number of steps**	170 (290)	233 (577)	472 (908)
**Median (interquartile range) time spent in sedentary activity (minutes)**	713 (20)	711 (28)	2133 (74)
**Median (interquartile range) time spent in moderate/vigorous activity (minutes)**	2 (6)	2 (8)	6 (15)
**Median (interquartile range) total energy expenditure (calories)**	851 (280)	878 (256)	2502 (738)
**Median (interquartile range) active energy expenditure (calories)**	6 (24)	10 (30)	23 (75)

### Outcomes associated with lower postoperative activity

Patients who took less than the median of 500 steps during the total early postoperative period (8 am Day 2-8 pm Day 3) (n=50) and demonstrated significantly lower step count (220 Vs 1128 steps, p < 0.001) than the remaining patients, lower median percentage active energy expenditure (active energy expenditure /total energy expenditure) (0% vs. 1%, p = 0.023), and lower median moderate intensity activity >3 METS (2 minutes vs. 10 minutes, p = 0.003). Twenty of these ‘lower activity’ patients had not been able to mobilise on postoperative day 1, compared to only 2 patients demonstrating ‘higher activity’ (p < 0.001). There was a significantly longer median postoperative LOS of 6 (3) days vs. 5 (2) days in those less active (p = 0.013), and a higher frequency of PPC at 20% (n = 10) vs 4% (n = 2) (p = 0.028).

### Perceived pain

**Figure 1:**
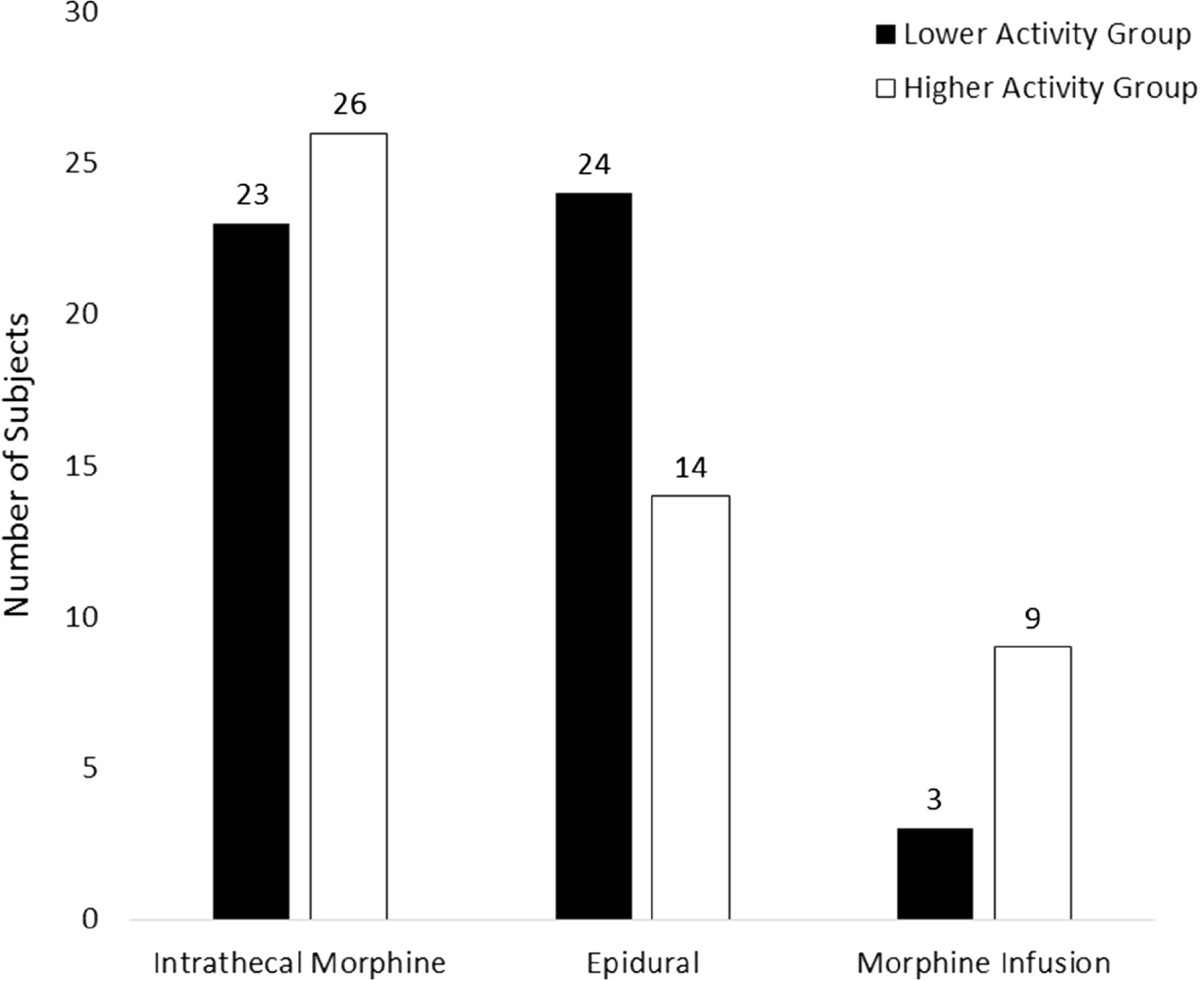
**Postoperative analgesia.**

On postoperative day 2 20 (40%) of those demonstrating lower activity had pain scores reflective of moderate or severe pain, compared to 6 (12%) demonstrating higher activity (p = 0.014), and on postoperative day 3 18 (36%) compared to 4 (8%) (p = 0.004). There was no significant difference (p = 0.103) in type of analgesia administered to these subjects, Figure 1.

### Factors predictive of lower postoperative activity

Differences in demographic and risk variables are shown in Table 2. Logistic regression was performed to identify factors predictive of lower postoperative activity. Independent variables entered into the regression model were those that demonstrated p-values <0.05 on univariate analysis (Table 2). Diffusing capacity was not routinely measured as guided by UK national guidelines at the time [15], therefore this factor was not entered into the regression model. The model correctly classified 50% of patients with lower activity. A significant contribution to the model was made by age ≥75 years (p = 0.012), predicted FEV_1_ < 70% (p = 0.033) and lower self reported preoperative activity (<level 6) (p = 0.019). The odds ratios for age (4.47, 1.38-14.45) predicted FEV_1_ (2.72, 1.08-6.81) and lower reported preoperative activity (3.73, 1.24-11.25) indicated these variables to be predictors of lower postoperative activity.

**Table 2:** **Demographic and risk variables**

Demographics and risk factors	Lower activity patients (n = 50)	Higher activity patients (n = 49)	p value
**Male**	54% (25)	46% (21)	0.476
**Lung cancer**	88% (44)	98% (48)	0.059
**RCT intervention group**	48% (24)	55% (27)	0.480
**% predicted FEV**_**1**_ **mean (±SD)**	70 (15)	80 (21)	0.006
**ppoFEV**_**1**_ **mean (±SD)**	57 (16)	64 (22)	0.042
**Age (years) median (interquartile range)**	71 (11)	66 (12)	0 .014
**BMI mean (±SD)**	26 ± 5	26 ± 4	0.940
**ASA ≥ 3**	57% (35)	43% (26)	0.083
**Current smoking/ex smokers of up to 6 weeks**	55% (12)	46% (10)	0.667
**COPD**	67% (18)	33% (9)	0.042
**Preoperative activity level <2 km**	36% (18)	12% (6)	0.006

### Factors predictive of PPC

A further logistic regression was performed to ensure none of the factors independently associated with reduced postoperative physical activity were themselves associated with the development of PPC. The model correctly classified 88% of patients with development of PPC. A significant contribution to the model was made by COPD (p = 0.001), and the odds ratio (11.33, 2.78-46.24) confirmed COPD alone to be a predictor of PPC.

## Discussion

This study defines the extent of limitation on physical activity immediately following major thoracic surgery. Mean daily step count during the early postoperative period was markedly reduced at only 3% of the preoperative mean (8654 steps) of a similar group of individuals [5] awaiting surgery. Postoperative activity has also been shown to be very limited at one month after surgery, with a reduction in step count of 25% and 49% observed in lobectomy and pneumonectomy patients respectively [7].

Overall LOS was comparable to that previously recorded for similar patients [4, 18], but those who were less active had significantly longer LOS. The data collected cannot confirm that reduced activity causes PPC, or vice versa, and the influence of other factors such as pain, which has previously been associated with prolonged LOS [19], cannot be ruled out as a contributing factor. Postoperative care pathways including early mobilisation have previously been shown to improve outcomes [3], but further randomised studies would be required to determine the specific effect of physical activity alone on frequency of PPC or LOS.

Several preventable or modifiable factors were observed to limit postoperative activity in this study, with pain determined as significantly higher in those least active. The importance of effective pain relief in facilitating early mobilisation and better outcome has been previously been identified [8] and strategies to improve pain relief are highly relevant in developing postoperative care pathways. A relatively large number of patients were unable to mobilise on postoperative day 1 and were then less active on postoperative day 2 and 3. Causes included pain, dizziness secondary to low blood pressure, continuous intercostal drain suction, drowsiness, vomiting and nausea.

Some of the limitations observed could be addressed by postoperative care pathways including widespread use of paravertebral rather than epidural cathethers which are associated with lower early mobility and overall more complications [20]. The use of digital chest drains with portable suction which ‘free the patient’ should also be considered [21]. All patients in this study were managed with 1 or 2 intercostal drains and a urinary catheter following surgery, as well as varying analgesic attachments. These types of surgical attachments have been shown to reduce patient ‘uptime’ [8]. Avoiding or early removal of these types of attachments is an integral part of enhanced recovery pathways applied widely in the UK across colorectal, musculoskeletal, urological and gynaecological surgery [22]; this type of philosophy needs to be adopted following all types of major surgery. Assistance to mobilise has been shown to be required until postoperative day 3 following major abdominal surgery [8], with ‘uptime’ greater at times when more staff were available. Given the low levels of postoperative activity observed in this study, increased assistance could be of benefit, specifically in the elderly or for those with poor preoperative activity, both factors being predictive of limitation.

Independent factors predictive of lower postoperative activity included age ≥75 years, predicted FEV_1_ < 70% and lower self reported preoperative activity. Limited preoperative exercise tolerance may be caused by reduced cardiopulmonary function, which in combination with diffusion tests is predictive of postoperative outcome [23] or, by musculoskeletal issues which limit ability to engage in the exercise tests. Whether cardiopulmonary or musculoskeletal, both causes of reduced activity indicate poor function which is a poor prognostic factor. Pulmonary rehabilitation may be of benefit in improving general physical activity preoperatively; improved exercise capacity has been shown prior to surgery in both patients with [24] and without COPD [25, 26] as well as improvements in quantity of daily activity [27]. If a patient is in a preoperative culture of mobilisation and exercise it seems reasonable to hypothesise that they will be more likely to engage in this type of activity following surgery [28]. Certainly preoperative programmes, including exercise, for those at high risk have translated into improvement in postoperative outcomes [29]. Preoperative education including contact with relevant physiotherapists and surgeons is of importance to maximising mobility; if a patient and their relatives understand what is required of them and why, success is more likely [3].

The present study could have been improved by measuring preoperative activity in the subjects observed to establish the exact impact of surgery in this group. A significant difference in activity between lobectomy and pneumonectomy patients has been previously demonstrated [6] but with only 10 subjects undergoing pneumonectomy in our study the effect of type of surgery on activity in the immediate period following surgery was not investigated. We acknowledge the growing trend towards minimally invasive surgery for lobectomy, but effects on early postoperative physical activity following this are yet to be determined. In the foreseeable future many patients will undergo thoracotomy for major lung resection, and our results will remain applicable.

The patients studied were aware of the physical activity data collection as they were wearing monitors; this may have influenced how much activity was undertaken, although this is unlikely given the very low level of activity observed, also there was no visual display on the device so subjects were blinded to data output. Patients unable to wear monitors included those who were deemed too unwell or those being discharged from hospital on postoperative day 3. These patients may have represented the extremes of higher and lower activity, and their exclusion may have adversely affected results.

## Conclusion

We have defined the marked limitation in physical activity in the immediate postoperative following major thoracic surgery, showing that those with the poorest physical activity have increased LOS. Pain was significantly associated with limited physical activity in the early postoperative period, and many specific factors identified as preventing mobilisation on postoperative day 1 were also reversible/modifiable. Independent factors predictive of lower levels of postoperative physical activity included age, lung function and self reported preoperative activity level, the latter two potentially modifiable with preoperative physiotherapy/rehabilitation. Prompt assessment and recognition of these factors is needed in future, with timely and effective management incorporated into care pathways to maximise each patient’s potential to mobilise postoperatively.

## Authors’ original submitted files for images

Below are the links to the authors’ original submitted files for images. Authors’ original file for figure 1

## Abbreviations

ASA: American society of anaesthesiologists score
BMI: Body mass index
COPD: Chronic obstructive pulmonary disease
FEV_1_: Forced expiratory volume in one second
HDU: High dependency unit
LOS: Length of stay
MET: Metabolic equivalent of a task
PPC: Postoperative pulmonary complication.
