# Supplementary figures and images for: Potentially modifiable factors contribute to limitation in physical activity following thoracotomy and lung resection: a prospective observational study
Source: J Cardiothorac Surg. 2014 Sep 27;9:128. doi: 10.1186/1749-8090-9-128 (PMC4283127; doi:10.1186/1749-8090-9-128)

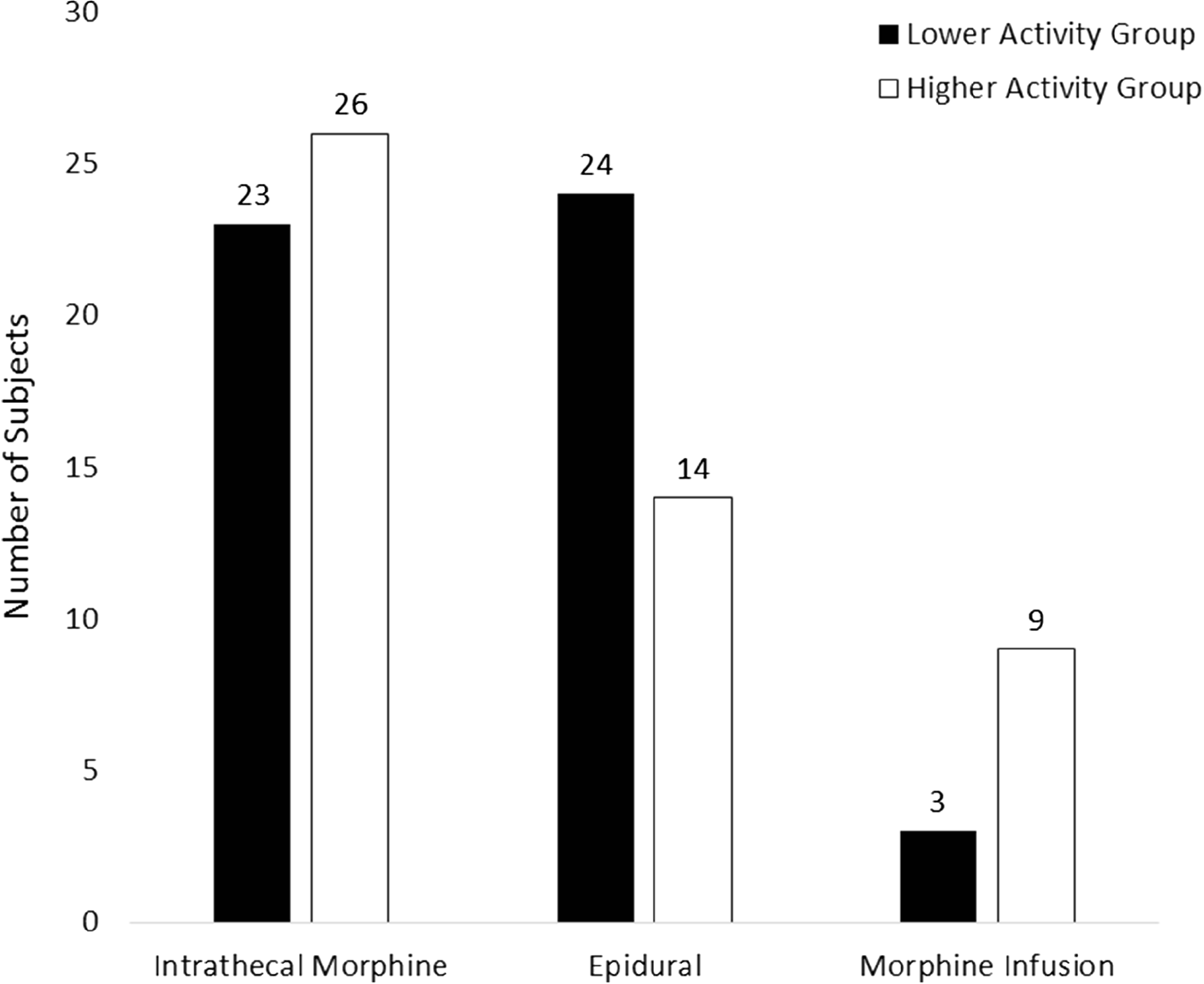

Supplement: Supplementary file 1 — Authors’ original file for figure 1 [file 13019_2013_1531_MOESM1_ESM.tiff]
